# Profile of Otorhinolaryngology-Related Emergency Department Visits and Revisits in a Tertiary Care Center in Riyadh, Saudi Arabia

**DOI:** 10.3390/healthcare14101378

**Published:** 2026-05-18

**Authors:** Othman Ibrahim Alabdalwahab, Fahad Waleed Bin Aziz, Abdulmohsen Jameel Alshammari, Mohammed Abdullah Bayounis, Bander Fahad Aljarallah, Jawad Yousef Alhabeeb, Hessah Ibrahim Alsarra, Muhnnad Abdulaziz Alghamdi, Meshary Binhotan, Abdullah Alshibani

**Affiliations:** 1 College of Medicine, King Saud bin Abdulaziz University for Health Sciences, Riyadh 11481, Saudi Arabia; othmanalabdalwahab@gmail.com (O.I.A.); binaziz518@gmail.com (F.W.B.A.); abdulmohsens.2001@gmail.com (A.J.A.); mohammedbayounis@gmail.com (M.A.B.); banderaljarallah0@gmail.com (B.F.A.); ajawad445@gmail.com (J.Y.A.); hessah.alsarra@gmail.com (H.I.A.); 2 King Abdullah International Medical Research Center, Riyadh 11481, Saudi Arabia; hotanm@ksau-hs.edu.sa; 3 Ministry of National Guard Health Affairs, Riyadh 14611, Saudi Arabia; 4 College of Medicine, King Saud bin Abdulaziz University for Health Sciences, Jeddah 22384, Saudi Arabia; muhnnad297@gmail.com; 5 King Abdullah International Medical Research Center, Jeddah 22384, Saudi Arabia; 6 Emergency Medical Services Department, College of Applied Medical Sciences, King Saud bin Abdulaziz University for Health Sciences, Riyadh 11481, Saudi Arabia; 7 Department of Emergency Medicine, King Abdulaziz Medical City, Ministry of National Guard Health Affairs, Riyadh 14611, Saudi Arabia

**Keywords:** ENT, otolaryngology, emergency, urgent, visit, revisit

## Abstract

**Background/Objectives**: Otorhinolaryngologic (ENT) complaints constitute a substantial proportion of emergency department (ED) visits, ranging from minor to life-threatening problems. The patterns and frequency of these presentations in Saudi Arabia remain poorly described. This study, therefore, aimed to address this gap by examining the most frequent ENT-related presentations to the ED at a tertiary center in Riyadh, Saudi Arabia, to identify common diagnoses, describe patient demographics, and evaluate annual trends in ED visits. **Methods**: A retrospective study of all consecutive ENT-related emergency department visits between January 2019 and December 2024 was conducted at King Abdulaziz Medical City, a tertiary care center in Riyadh, Saudi Arabia. Diagnosis classification used the International Classification of Diseases, 10th Revision (ICD-10). Data on patient demographics, presenting complaints, and hospital admissions were extracted. Descriptive analyses were performed to assess annual trends and common diseases. **Results**: A total of 22,014 patients were included in the present study, with a slight male (54%) predominance. Across 34,290 ED visits, annual presentations were the highest in 2019 (23.9%) and lowest in 2020 (9.2%). Most patients were discharged from ED (91.9%). The most frequent diagnoses were acute pharyngitis (29.1%), acute tonsillitis (26.2%), and otitis media (22.3%). **Conclusions**: The study examined the otorhinolaryngology conditions most frequently encountered in an ED setting. The findings highlight a range of ENT disorders that are commonly seen in this setting. Recognizing these prevalent conditions and their patterns can improve ED physicians’ preparedness, triage, and management of patients presenting with ENT emergencies.

## 1. Introduction

Otorhinolaryngology emergencies in the emergency department (ED) represent a significant portion of the cases requiring urgent care [1]. Ear, nose, and throat (ENT)-related emergencies range from self-limiting disorders to life-threatening emergencies, where airway obstruction or uncontrolled bleeding can lead to rapid deterioration if not promptly managed [2]. ED visits related to ENT conditions differ by region, depending on healthcare access, environmental factors, and demographics.

ENT emergency patterns across geographical regions highlight the importance of region-specific epidemiological data to make informed decisions regarding staffing, training, and resource allocation in emergency rooms [3]. For example, an investigation of Medicare data revealed that the prevalence of epistaxis (nosebleeds) visits to the ED was higher among older adults, males, and Black individuals than among females and non-Hispanic Whites. Seasonal effects were also observed, as the number of visits was lower during the summer and higher during the winter [4].

Emergency department visits can differ from urgent conditions, such as viral upper respiratory tract infections and rhinosinusitis, which are often resolved by using symptomatic treatment like analgesia and antihistamines, to life-threatening conditions that require emergent interventions, such as severe epistaxis, facial fractures, and peritonsillar abscesses, which may require medical or surgical intervention [5]. The most frequently presenting ENT emergencies in the emergency department include epistaxis, foreign bodies in the ear, nose, and throat of patients, facial fractures, and trauma [6]. Additionally, most emergent ENT cases that may compromise the airway are conditions like epiglottitis, inhaled foreign body obstructing the airway, and Ludwig’s angina [7]. If not promptly recognized and treated, these conditions can rapidly progress to respiratory distress, leading to severe complications or even mortality.

A study analyzing 38,793 patients over a five-year period found that ENT emergencies accounted for approximately 7759 cases per year, averaging 21 cases per day [3]. Another study done in a Secondary hospital in Spain has found that ENT emergencies represent a significant burden on emergency departments, with an attendance rate of 450 per 1000 inhabitants per year. Moreover, the most common diagnoses were upper respiratory tract infections, tonsillopharyngitis, and dizziness/vertigo [8].

Several studies have mentioned the incidence of ENT related visits in the emergency department. For example, a study conducted in a tertiary care hospital in Saudi Arabia showed that out of 103,328 patients with less frequent ED visits (1–3 visits), 7659 (7.41%) were ENT related, and the frequent ED visits (4–13 visits) of 42,226 patients, 2552 (6.04%) were ENT related [9]. Additionally, a tertiary hospital study in Saudi Arabia involving 15,850 pediatric patients showed that surgical intervention was indicated in 1.2% of pediatric ENT emergencies, with foreign body emergencies representing 42% of operative cases [10].

Although international and regional data are available, differences in healthcare facilities, referral patterns, and population characteristics limit the extrapolation of results across different healthcare settings [11]. Recent work from European tertiary centers has similarly highlighted the substantial contribution of ENT cases to overall emergency department workload and the frequent presentation of low-severity diagnoses that could potentially be managed in primary care [12]. A 2023 study from a tertiary hospital reported that ENT presentations remain a common reason for ED attendance and emphasized the importance of strengthening primary care capacity for ENT conditions, while a 2025 cross-sectional analysis of approximately 6000 ENT cases in a tertiary ED in Crete, Greece, underscored that many ENT diagnoses have a low severity index and may be suitable for outpatient management rather than emergency care [13]. There is a lack of studies done regarding this topic especially in Saudi Arabia; therefore, this study aims to investigate the incidence of various diagnoses of Otorhinolaryngology-related visits to the emergency department at a tertiary hospital in Riyadh, Saudi Arabia. The findings from this research could provide valuable insights into the epidemiology of ENT emergencies in the local population, which is important to improve care strategies regarding different ENT cases.

## 2. Materials and Methods

### 2.1. Study Design, Settings, and Participants

This retrospective cohort study was conducted through a structured review of medical records for patients who presented to the Emergency Department (ED) between January 2019 and December 2024. The study used a purposive institutional sampling approach, selecting King Abdulaziz Medical City as the tertiary care center of interest, and then included all consecutive patients presenting to the ED with ENT-related conditions during the study period. As an observational study, data were extracted from electronic patient records using the “BestCare” system, the electronic medical record platform implemented at King Abdulaziz Medical City (KAMC).

The study was carried out in the Emergency Department of KAMC, Riyadh, Saudi Arabia. KAMC is one of the largest and most comprehensive tertiary healthcare institutions in the country. Established in 1982, it currently operates 1973 inpatient beds and employs approximately 8000 healthcare and allied medical professionals.

The study included all patients who presented to the ED with ENT-related conditions during the study period. Patients were excluded if essential demographic information (e.g., age or sex) was missing, or if documentation of the ENT-related emergency (e.g., nature or diagnosis of the condition) was incomplete.

### 2.2. Study Variables

Data was collected by members of the research team through systematic review of electronic medical records for eligible patients presenting between January 2019 and December 2024. Visiting history was examined between that time frame, and planned visits before or after this period were not recorded; thus, first visit dates may not represent the true first-ever visit for each patient. The outcome variable revisit was defined as repeat visits to the ED within the study period (2019–2024), and any visits before or after this period was not considered. A standardized data collection form was used to ensure consistency and accuracy.

In this study, key variables included patient demographics (age group and sex), emergency department visit characteristics (calendar year, month, day of the week, and check-in time), and hospital disposition (admitted versus discharged). The primary diagnosis for each visit was coded using the International Classification of Diseases, 10th Revision (ICD-10), and diagnoses were grouped into broader ICD-10 categories. Revisit status was defined as the number of ENT-related emergency department visits per patient during the study period, including a specific indicator for revisits within 72 h of discharge.

All extracted data were anonymized prior to analysis and securely stored on a password-protected university computer accessible only to authorized study investigators.

### 2.3. Statistical Analysis

Descriptive statistics were used to summarize demographic characteristics and emergency visit details (Table 1, Table 2 and Table 3). Categorical variables were presented as frequencies and percentages. Continuous variables were assessed for normality and were found to be non-normally distributed; therefore, they were summarized as medians with interquartile ranges (IQR).

Associations between categorical variables and revisit status (Table 4) were examined using Fisher’s Exact Test. Multivariable logistic regression analyses were conducted to identify predictors of emergency department revisits (Table 5). Firth’s penalized logistic regression model was employed due to rare outcomes and separation issues. A two-sided *p*-value < 0.05 was considered statistically significant. All statistical analyses were performed using RStudio (version 2024.9.1.394, Boston, MA, USA) with R version 4.4.2.

### 2.4. Ethics

Ethical approval was obtained from the Institutional Review Board of King Abdullah International Medical Research Center (KAIMRC), Riyadh, Saudi Arabia (Study Number: NRR25/074/3).

## 3. Results

### 3.1. Demographic Characteristics of Patients

**Figure 1:**
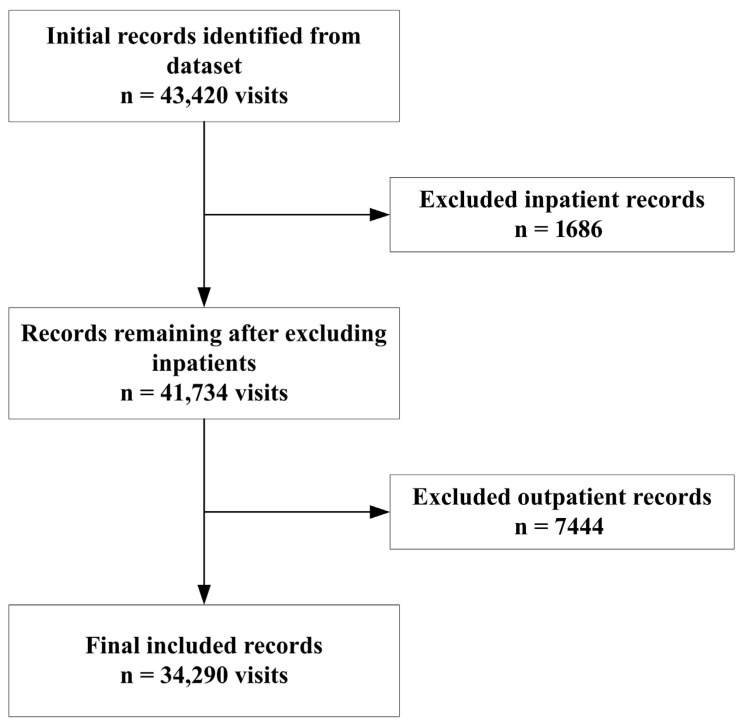
Flow diagram of study record selection.

A total of 43,420 records were initially identified, of which 34,290 emergency department visits, corresponding to 22,014 patients, met the inclusion criteria after excluding inpatient and outpatient records (Figure 1). The demographic characteristics of included patients are summarized in Table 1. Among 22,014 patients, 54.0% were male and 46.0% were female. The majority of patients were younger than 18 years (79.0%), while 16.0% were aged 18 to 55 years, and only 5.0% were older than 55 years (Table 1).

**Table 1:** Demographic characteristics of patients (N = 22,014).

Characteristic	Overall N = 22,014, N (%)	Pediatric (<18 Years) N = 17,391, N (%)	Adult (≥18 Years) N = 4623, N (%)
Gender			
Male	11,886 (54.0%)	9673 (55.6%)	2213 (47.9%)
Female	10,128 (46.0%)	7718 (44.4%)	2410 (52.1%)
Age (years)			
<18	17,391 (79.0%)	17,391 (100.0%)	0 (0.0%)
18 to 55	3524 (16.0%)	0 (0.0%)	3524 (76.2%)
>55	1099 (5.0%)	0 (0.0%)	1099 (23.8%)

### 3.2. Characteristics of ER Visits

**Figure 2:**
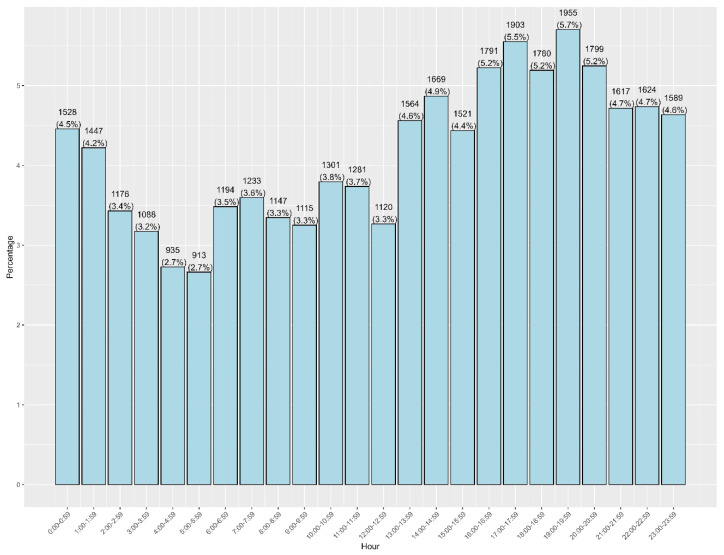
Hourly distribution of frequency and trend of ENT visits over study period (34,290 visits).

**Figure 3:**
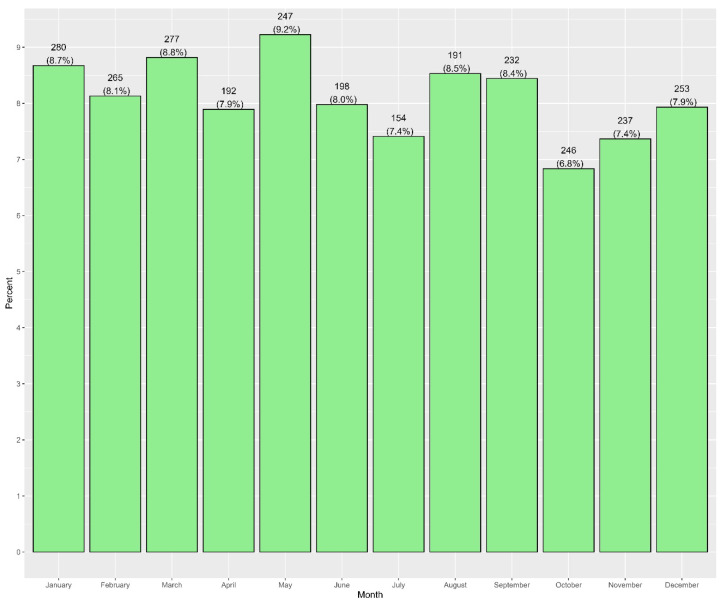
Monthly distribution of hospital admissions among ENT-related emergency department visits during the study period (N = 2772).

Characteristics of emergency department visits are presented in Table 2. Across 34,290 emergency department visits, the highest proportion occurred in 2019 (23.9%), followed by 2023 (21.5%) and 2024 (17.7%). October had the highest monthly visit rate (10.5%), with relatively even distributions across days of the week. The most frequent check-in time was during the night shift (21:00 to 5:59), accounting for 34.8% of visits. Most patients were discharged (91.9%), while 8.1% were admitted (Table 2). Among those admitted, 64.5% were admitted to the ENT department, followed by 17.8% to the Pediatric Inpatient Team (General, Table S1 in the Supplementary File). The frequency of emergency department visits was lowest in the early morning (03:00–06:59; each ~2.7–3.5%), rises steadily from late morning, and accelerates after 14:00. The peak occurs at 19:00–19:59 (1955; 5.7%), with a broad evening plateau spanning 17:00–20:59 (≈5.2–5.7% per hour, Figure 2). Of all ED visits, 2772 visits were admitted to hospital: more commonly in the month of May (Figure 3). In each year from 2019 to 2024, over 80% of patients had a single ER visit. Revisit rates ranged from 11.5% to 18.1%, with the highest proportion of repeat visits observed in 2023 (18.1%) and 2024 (18.0%, Table S2 in the Supplementary File).

**Table 2:** Characteristics of ER visits (N = 34,290).

Characteristic	Overall N = 34,290 ^1^	Pediatric (<18 Years) N = 28,643 ^1^	Adult (≥18 Years) N = 5647 ^1^
Patient revisited the ER after 72 h discharge from index admission			
No	33,629 (98.1%)	28,116 (98.2%)	5513 (97.6%)
Yes	661 (1.9%)	527 (1.8%)	134 (2.4%)
Year of visit			
2019	8180 (23.9%)	6923 (24.2%)	1257 (22.3%)
2020	3165 (9.2%)	2398 (8.4%)	767 (13.6%)
2021	3503 (10.2%)	2673 (9.3%)	830 (14.7%)
2022	5988 (17.5%)	5199 (18.2%)	789 (14.0%)
2023	7371 (21.5%)	6330 (22.1%)	1041 (18.4%)
2024	6083 (17.7%)	5120 (17.9%)	963 (17.1%)
Month of visit			
January	3228 (9.4%)	2719 (9.5%)	509 (9.0%)
February	3259 (9.5%)	2813 (9.8%)	446 (7.9%)
March	3141 (9.2%)	2685 (9.4%)	456 (8.1%)
April	2432 (7.1%)	2003 (7.0%)	429 (7.6%)
May	2677 (7.8%)	2224 (7.8%)	453 (8.0%)
June	2482 (7.2%)	2007 (7.0%)	475 (8.4%)
July	2078 (6.1%)	1596 (5.6%)	482 (8.5%)
August	2239 (6.5%)	1781 (6.2%)	458 (8.1%)
September	2748 (8.0%)	2285 (8.0%)	463 (8.2%)
October	3599 (10.5%)	3105 (10.8%)	494 (8.7%)
November	3218 (9.4%)	2724 (9.5%)	494 (8.7%)
December	3189 (9.3%)	2701 (9.4%)	488 (8.6%)
Day of visit			
Saturday	5192 (15.1%)	4347 (15.2%)	845 (15.0%)
Sunday	4955 (14.5%)	4140 (14.5%)	815 (14.4%)
Monday	4705 (13.7%)	3928 (13.7%)	777 (13.8%)
Tuesday	4911 (14.3%)	4087 (14.3%)	824 (14.6%)
Wednesday	4794 (14.0%)	3997 (14.0%)	797 (14.1%)
Thursday	4692 (13.7%)	3885 (13.6%)	807 (14.3%)
Friday	5041 (14.7%)	4259 (14.9%)	782 (13.8%)
ER Check-in Time			
Morning (06:00 to 11:59)	7271 (21.2%)	5942 (20.7%)	1329 (23.5%)
Afternoon (12:00 to 16:59)	7665 (22.4%)	6343 (22.1%)	1322 (23.4%)
Evening (17:00 to 20:59)	7437 (21.7%)	6321 (22.1%)	1116 (19.8%)
Night (21:00 to 5:59)	11,917 (34.8%)	10,037 (35.0%)	1880 (33.3%)
Length of Stay (incl. ER)	1.0 (1.0–1.0)	1.0 (1.0–1.0)	1.0 (1.0–1.0)
Length of Stay in Time (incl. ER)	0.1 (0.1–0.2)	0.1 (0.1–0.1)	0.2 (0.1–0.2)
Disposition			
Admitted	2772 (8.1%)	2199 (7.7%)	573 (10.1%)
Discharged	31,518 (91.9%)	26,444 (92.3%)	5074 (89.9%)

^1^ N (%); Median (Q1–Q3).

### 3.3. Frequencies and Percentages of Visits by ICD-10 Codes and ICD-10 Code Categories

The distribution of diagnoses among emergency department visits is presented in Table 3. Among the 34,290 visits, the most common diagnosis was acute pharyngitis: unspecified (29.1%); followed by acute tonsillitis: unspecified (26.2%); and otitis media: unspecified (22.3%). Other frequently recorded diagnoses included epistaxis (5.3%); otitis externa: unspecified (2.7%); and acute otitis media (1.8%, Table 3). All other diagnoses accounted for less than 1% each (Table S3).

When grouped by ICD-10 code categories, the most common diagnoses were within the J category (diseases of the respiratory system), comprising 60.3% of cases, followed by H codes (diseases of the eye, adnexa, ear, and mastoid process) at 29.4%. Other categories each accounted for less than 7% of diagnoses (Table S4).

**Table 3:** Frequencies and percentages of visits by ICD-10 codes reported in at least 1% of participants.

Characteristic	Overall N = 34,290 ^1^	Pediatric (<18 Years) N = 28,643 ^1^	Adult (≥18 Years) N = 5647 ^1^
Internal Diagnosis			
Acute otitis media	621 (1.8%)	567 (2.0%)	54 (1.0%)
Acute pharyngitis—unspecified	9980 (29.1%)	9202 (32.1%)	778 (13.8%)
Acute tonsillitis—unspecified	8971 (26.2%)	7699 (26.9%)	1272 (22.5%)
Epistaxis	1830 (5.3%)	825 (2.9%)	1005 (17.8%)
Otitis externa—unspecified	914 (2.7%)	399 (1.4%)	515 (9.1%)
Otitis media—unspecified	7654 (22.3%)	7136 (24.9%)	518 (9.2%)
Sinusitis	559 (1.6%)	220 (0.8%)	339 (6.0%)

^1^ N (%).

### 3.4. Baseline Characteristics of Patients Who Visited Once Versus Those Who Visited Twice or More

A comparison of baseline characteristics between patients with a single visit and those with multiple visits during the study period is presented in Table 4. Significant differences were found between patients with a single visit and those with multiple visits across several characteristics. Patients with multiple visits were more likely to be male (56.2% vs. 53.0%, *p* < 0.001), under 18 years (89.6% vs. 74.3%, *p* < 0.001), and have been admitted during their initial visit (8.7% vs. 4.3%, *p* < 0.001). Recurrent visitors were also more likely to have a shorter length of stay (<4 h: 79.5% vs. 74.9%, *p* < 0.001). ICD-10 code distributions differed significantly (*p* < 0.001), with higher proportions of H and J code diagnoses among recurrent visitors (Table 4). Monthly visit distributions also varied significantly between groups (*p* < 0.001, Table S5). No significant differences were found by day of visit (*p* = 0.706) or ER check-in time (*p* = 0.068, Table S5).

**Table 4:** Baseline characteristics of patients who visited once versus those who visited twice or more (N = 22,014).

Characteristic	Once N = 15,293 ^1^	Twice or More N = 6721 ^1^	*p*-Value ^2^
Year of visit			<0.001
2019	4064 (61.7%)	2518 (38.3%)	
2020	1456 (66.5%)	732 (33.5%)	
2021	1608 (66.5%)	810 (33.5%)	
2022	2516 (67.9%)	1187 (32.1%)	
2023	3000 (74.6%)	1019 (25.4%)	
2024	2649 (85.3%)	455 (14.7%)	
Sex			<0.001
Male	8112 (68.2%)	3774 (31.8%)	
Female	7181 (70.9%)	2947 (29.1%)	
Age			<0.001
<18	11,367 (65.4%)	6024 (34.6%)	
18 to 55	3024 (85.8%)	500 (14.2%)	
>55	902 (82.1%)	197 (17.9%)	
First Disposition			<0.001
Admitted	650 (52.8%)	582 (47.2%)	
Discharged	14,643 (70.5%)	6139 (29.5%)	
LOS (hrs)			<0.001
<4	11,454 (68.2%)	5346 (31.8%)	
≥4	3839 (73.6%)	1375 (26.4%)	
LOS (day)			0.597
≤1	15,279 (69.5%)	6718 (30.5%)	
2	13 (81.3%)	3 (18.8%)	
≥3	1 (100.0%)	0 (0.0%)	
ICD			<0.001
A	1 (33.3%)	2 (66.7%)	
B	1 (100.0%)	0 (0.0%)	
D	0 (NA%)	0 (NA%)	
F	0 (0.0%)	2 (100.0%)	
H	4313 (67.4%)	2087 (32.6%)	
J	9028 (69.1%)	4032 (30.9%)	
K	0 (NA%)	0 (NA%)	
M	0 (0.0%)	1 (100.0%)	
R	1277 (76.3%)	397 (23.7%)	
S	23 (82.1%)	5 (17.9%)	
T	650 (76.9%)	195 (23.1%)	
Z	0 (NA%)	0 (NA%)	

^1^ N (%). ^2^ Pearson’s Chi-squared test; Fisher’s exact test.

### 3.5. Multivariate Logistic Regression of Predictors of Revisits

Multivariable logistic regression analysis was conducted to determine independent predictors of emergency department revisits within the study period. In the adjusted model, female sex remained associated with reduced odds of revisit (OR = 0.93, 95% CI, 0.88 to 0.99, *p* = 0.015). Patients aged 18 to 55 (OR = 0.31, 95% CI, 0.28 to 0.34, *p* < 0.001) and >55 (OR = 0.41, 95% CI, 0.35 to 0.49, *p* < 0.001) had lower odds of revisit than those under 18. Discharged patients were less likely to revisit (OR = 0.41, 95% CI, 0.36 to 0.46, *p* < 0.001). A length of stay ≥4 h was associated with reduced odds of revisit (OR = 0.91, 95% CI, 0.84 to 0.98, *p* = 0.010). Compared to January, visits in June (OR = 0.82, 95% CI, 0.71 to 0.94, *p* = 0.005), August (OR = 0.84, 95% CI, 0.72 to 0.97, *p* = 0.020), September (OR = 0.77, 95% CI, 0.67 to 0.88, *p* < 0.001), October (OR = 0.76, 95% CI, 0.67 to 0.86, *p* < 0.001), November (OR = 0.72, 95% CI, 0.63 to 0.82, *p* < 0.001), and December (OR = 0.68, 95% CI, 0.59 to 0.78, *p* < 0.001) remained significantly associated with lower odds of revisit (Table 5).

**Table 5:** Multivariate logistic regression of predictors of revisits.

Characteristic	All Time			COVID Period (2020–2022)		
	OR ^1^	95% CI	*p*-Value	OR ^1^	95% CI	*p*-Value
Sex						
Male	Reference	Reference		Reference	Reference	
Female	0.93	0.88, 0.99	0.015	0.97	0.88, 1.07	0.539
Age						
<18	Reference	Reference		Reference	Reference	
18 to 55	0.31	0.28, 0.34	<0.001	0.28	0.24, 0.33	<0.001
>55	0.41	0.35, 0.49	<0.001	0.43	0.34, 0.53	<0.001
First Disposition						
Admitted	Reference	Reference		Reference	Reference	
Discharged	0.41	0.36, 0.46	<0.001	0.38	0.31, 0.45	<0.001
LOS (hrs)						
<4	Reference	Reference		Reference	Reference	
≥4	0.91	0.84, 0.98	0.010	0.93	0.83, 1.04	0.201
Month of visit						
January	Reference	Reference		Reference	Reference	
February	1.00	0.88, 1.13	0.953	0.89	0.71, 1.10	0.278
March	1.11	0.98, 1.27	0.096	1.08	0.87, 1.35	0.473
April	0.97	0.84, 1.11	0.668	1.07	0.84, 1.36	0.574
May	0.94	0.82, 1.08	0.394	1.09	0.87, 1.36	0.450
June	0.82	0.71, 0.94	0.005	0.76	0.61, 0.95	0.018
July	1.04	0.90, 1.21	0.560	1.08	0.85, 1.37	0.524
August	0.84	0.72, 0.97	0.020	0.89	0.71, 1.13	0.348
September	0.77	0.67, 0.88	<0.001	0.79	0.63, 0.99	0.039
October	0.76	0.67, 0.86	<0.001	0.79	0.63, 0.98	0.031
November	0.72	0.63, 0.82	<0.001	0.82	0.66, 1.02	0.074
December	0.68	0.59, 0.78	<0.001	0.84	0.67, 1.06	0.138
ER Check-in Time						
Morning (06:00 to 11:59)	Reference	Reference		Reference	Reference	
Afternoon (12:00 to 16:59)	1.01	0.92, 1.10	0.885	0.87	0.75, 1.01	0.059
Evening (17:00 to 20:59)	1.07	0.98, 1.17	0.154	0.96	0.83, 1.11	0.562
Night (21:00 to 5:59)	1.05	0.97, 1.14	0.238	0.99	0.87, 1.13	0.867

^1^ Results are based on an univariable Firth’s penalized logistic regression models. Abbreviation: CI = Confidence Interval.

## 4. Discussion

In this large tertiary care study of ENT-related emergency visits in Riyadh, we found that pediatric patients constituted the vast majority (79%) of cases, as shown in Table 1. Common upper respiratory infections dominated the diagnostic spectrum, with acute pharyngitis (29.1%) and acute tonsillitis (26.2%) together accounting for over half of all ENT emergencies. Otologic infections were also frequent, as otitis media (unspecified) comprised 22.3% of visits; these diagnostic distributions are summarized in Table 4 and ICD-10 category groupings in Table 5. By contrast, classic ENT emergencies such as epistaxis made up a smaller yet notable fraction (5.3%). The distribution of visits across years, months, and days, and the predominance of single visits per patient are detailed in Table 2 and Table 3. The distribution of visits was fairly even across weekdays and months, although October showed a slight peak (~10.5% of annual visits). A sharp decline in visits was observed in 2020–2021, followed by a rebound in 2022–2023, reflecting the disruptive impact of the COVID-19 pandemic on healthcare-seeking behavior. Notably, presentation timing skewed heavily toward evenings and nights, with over one-third of visits occurred during the night shift with activity peaking in the late evening hours (around 19:00–20:00). This temporal pattern suggests that many patients seek ENT emergency care after routine clinic hours. Most encounters were managed non-operatively and discharged (91.9%), while only 8.1% required hospital admission (predominantly to the ENT service). The low 72 h revisit rate (1.9%) indicates that initial management was generally effective, with relatively few patients returning for unresolved issues.

Beyond the overall low 72 h revisit rate, several patient and visit characteristics were associated with repeat presentations. In the multivariable Firth’s logistic regression model (Table 5), younger age, male sex, prior admission, and longer length of stay during the index visit were independently associated with higher odds of revisiting, whereas adult age groups and shorter stays were associated with reduced revisit risk. These findings suggest that children, particularly boys with more complex or prolonged initial encounters, may benefit from targeted discharge counseling and closer follow-up to minimize avoidable returns to the ED.

Overall, these findings portray a high-volume ENT emergency service driven largely by pediatric infections and minor ENT ailments, with a smaller proportion of true emergencies requiring admission.

### 4.1. Epidemiology of ENT Emergencies

Our findings align with and expand upon prior epidemiological studies of ENT emergencies, while also highlighting important differences attributable to population demographics and healthcare context. The predominance of pediatric upper respiratory infections (pharyngitis, tonsillitis) in our cohort mirrors the experience of other centers serving a large pediatric population. For example, a U.S. nationwide ED analysis (2009–2011) found that 62.7% of patients presenting with otologic complaints were children under 18 years [14]. Otitis media was the single most common ED otolaryngologic diagnosis in that analysis, consonant with our result that otitis media (acute and unspecified) was a leading reason for ED visits. Many of these cases in the U.S. were treated and released, reflecting their generally benign, non-emergent nature. Our hospital similarly managed most ENT cases on an outpatient basis, which is reassuring and suggests consistency in practice worldwide.

### 4.2. Diagnoses of ENT Emergencies

However, studies from centers with older patient demographics report different profiles of ENT emergencies. In an Indian tertiary hospital, epistaxis was the most common ENT emergency (25.6%), far exceeding any single diagnosis in our series [3]. Likewise, a recent prospective study in Greece with a predominantly adult population noted that the most frequent ED diagnoses were external ear infections (6.9%) and epistaxis (6.7%), while acute pharyngitis comprised only 3.6% of cases [12]. This contrasts sharply with our pediatric-dominated cohort, where pharyngitis alone was nearly one-third of visits. The Greek study also reported a 6.2% hospital admission rate—comparable to our 8.1%—with peritonsillar abscess being the top cause for inpatient ENT care [13]. Such differences illustrate how age distribution and referral patterns shape the spectrum of ENT emergencies: centers serving more adults tend to see relatively more epistaxis, otologic disease, and abscesses, whereas pediatric-heavy settings see more airway infections and tonsillitis [2]. Epistaxis accounted for 5.3% of ENT-related visits in our cohort. Although this proportion is lower than that reported in many adult-oriented series, it remains clinically significant [2]. Globally, epistaxis is recognized as a common reason for seeking emergency care, representing approximately one in every 200 ED visits in the United States [15]. The comparatively lower proportion in our study likely reflects the younger age distribution of our population, as epistaxis is more prevalent among older adults [2]. Nonetheless, our findings correspond with international reports identifying epistaxis as a recurrent and prominent ENT emergency across different healthcare settings [2].

### 4.3. Impact of COVID-19 on the Presentation of ENT Emergencies

Another key point is the impact of the COVID-19 pandemic on ENT emergency trends. Our yearly visit totals dropped by over 50% in 2020 compared to 2019, then gradually rose back close to pre-pandemic levels by 2023. This pattern is consistent with international observations reported in other otolaryngology emergency departments during the COVID-19 pandemic [16,17]. A systematic review and meta-analysis of otolaryngologic ED visits noted a significant overall decline in ENT presentations during the lockdown period (only ~31.5% of pre-lockdown volume), accompanied by a shift in case mix: fewer infectious emergencies (e.g., tonsillitis) and relatively more non-infectious problems like epistaxis, foreign bodies, and airway issues after lockdown [16,18]. Our data reflect a similar trend, with the sharp decline in 2020–21 corresponding to periods of social distancing and reduced care-seeking for routine infections, while the rebound by 2023 suggests a return to typical infectious case volumes, consistent with recent literature [19]. Moreover, the seasonal and time-of-day distributions in our study (e.g., peak in autumn, evening surge) were likely influenced by behavioral changes during and after the pandemic, as also suggested in the literature [20]. Taken together, these findings align with global reports on pandemic related shifts in ENT emergencies and add important region-specific insights from Saudi Arabia that were previously limited [20].

### 4.4. Discharge Disposition of ENT Emergencies

Our findings demonstrate that the majority of ENT-related ED visits represent low-acuity infections that can be effectively managed in outpatient or primary care settings [13,21]. With over 90% of patients discharged home, there is clear potential to reduce emergency department burden through expanded access to after-hours primary care, urgent care clinics, and telemedicine triage programs [22]. International data similarly indicate that many common ENT presentations have low hospitalization rates and can be safely redirected away from the ED with appropriate triage and follow-up systems [14,23]. Strengthening these pathways would not only improve resource use but also allow emergency services to focus on the smaller subset of true ENT emergencies such as airway compromise and deep neck infections that require immediate specialist intervention [13]. In Saudi Arabia, the clinical implications extend further due to the pediatric predominance and the pronounced evening surge in visits [24]. These patterns signal the need for enhanced pediatric ENT training among emergency clinicians and improved availability of ENT consultation during peak hours [25]. Establishing extended-hours ENT clinics or telehealth services could alleviate nighttime ED crowding by offering timely assessment for non-urgent conditions [26]. Resource planning should also account for seasonal increases in ENT presentations during cooler months [27]. Additionally, certain conditions such as epistaxis in older adults with cardiovascular comorbidities emphasize the importance of integrated follow-up between ENT, primary care, and chronic disease services to prevent recurrent emergency visits [28,29]. Collectively, these implications highlight opportunities to optimize ENT emergency care both nationally and internationally through better triage, improved outpatient access, and multidisciplinary continuity of care [30].

### 4.5. Strengths and Limitations

This study has several notable strengths. It represents one of the largest analyses of ENT-related emergency visits in the Middle East, encompassing more than 34,000 encounters across six years. The use of a comprehensive electronic database with standardized ICD-10 coding allowed for detailed characterization of diagnoses, admissions, and revisits. The multi-year design enabled evaluation of temporal trends, including the impact of the COVID-19 pandemic, offering insights not captured by single-year studies. Conducted in a tertiary care setting with full ENT specialty support, the study provides a reliable benchmark for similar institutions, and the low loss to follow-up further strengthens the accuracy of revisit estimates.

Nonetheless, several limitations should be acknowledged. As a single-center study in an urban tertiary hospital, the findings may not fully generalize to smaller or rural facilities with different case mixes or referral patterns. The high pediatric volume at our institution may also under-represent adult ENT emergencies, limiting comparisons with hospitals serving broader demographics. Retrospective reliance on ICD-10 coding introduces the possibility of misclassification or under-capture of certain presentations, especially when symptoms were coded under non-specific categories. Additionally, the study did not assess detailed management decisions or long-term outcomes beyond 72 h revisits, nor did it evaluate patient factors influencing ED utilization. External influences such as lockdowns or seasonal viral trends were also not formally modeled. These limitations should be considered when interpreting the findings.

## 5. Conclusions

This study offers a detailed analysis of ENT-related presentations to the ED at a major tertiary hospital in Riyadh, Saudi Arabia. The findings showed that pediatric upper respiratory and ear infections constitute the majority of visits, whereas true emergencies requiring admission remain relatively uncommon. Factors such as younger age, male sex, prior admission, and longer duration of stay were associated with increased likelihood of repeat visits. These results highlight the need for accessible after-hours primary care and urgent ENT clinics, more structured triage systems that can safely redirect low-acuity ENT cases away from the ED, and focused management strategies for high-acuity emergencies. Conducting similar studies throughout the region could improve insight into ENT emergencies and revisit patterns, improving patient care and outcomes.

## Data Availability

The raw data supporting the conclusions of this article will be made available by the principal investigator upon request.

## Supplementary Materials

The following supporting information can be downloaded at: https://www.mdpi.com/article/10.3390/healthcare14101378/s1, Table S1: Characteristics of admission departments (n = 34,290); Table S2: Number of visits per year (n = 27,902); Table S3: Frequencies and percentages of visits by ICD-10 codes; Table S4: Frequencies and percentages of visits by ICD-10 codes (n = 34,290); Table S5: Baseline characteristics of patients who visited once versus those who visited twice or more (n = 22,014).

## Abbreviations

The following abbreviations are used in this manuscript:

ED	Emergency department
ENT	Ear, nose, throat/otorhinolaryngology
ICD-10	International Classification of Diseases, 10th Revision
