# Supplementary material for: Profile of Otorhinolaryngology-Related Emergency Department Visits and Revisits in a Tertiary Care Center in Riyadh, Saudi Arabia
Source: Healthcare (Basel). 2026 May 18;14(10):1378. doi: 10.3390/healthcare14101378 (PMC13206701; doi:10.3390/healthcare14101378)
Supplement: Supplementary file 1 [file healthcare-14-01378-s001.zip › healthcare-4194966-supplementary.pdf]

Table S1. Characteristics of admission departments (n=34290).

**Characteristic**	**Overall** N = 34,290[1]	**Pediatric (<18 years)** N=28643[1]	**Adult (≥18 years)** N=5647[1]
Admission Dept			
Cardiology	2 (0.1%)	1 (0.1%)	1 (0.2%)
ENT	1,087 (64.5%)	695 (57.9%)	392 (80.8%)
Gastroenterology	15 (0.9%)	1 (0.1%)	14 (2.9%)
General pediatrics	82 (4.9%)	82 (6.8%)	0 (0.0%)
Genetics and Precision Medicine	2 (0.1%)	2 (0.2%)	0 (0.0%)
Hematology	2 (0.1%)	0 (0.0%)	2 (0.4%)
Hepatology	1 (0.1%)	0 (0.0%)	1 (0.2%)
Internal Medicine	63 (3.7%)	1 (0.1%)	62 (12.8%)
Medical Oncology	4 (0.2%)	0 (0.0%)	4 (0.8%)
Neurology	4 (0.2%)	0 (0.0%)	4 (0.8%)
Oral & Maxillofacial Surgery	3 (0.2%)	0 (0.0%)	3 (0.6%)
Pediatric Cardiology	7 (0.4%)	7 (0.6%)	0 (0.0%)
Pediatric Dental Surgery	5 (0.3%)	5 (0.4%)	0 (0.0%)
Pediatric ENT	35 (2.1%)	35 (2.9%)	0 (0.0%)
Pediatric Gastroenterology	24 (1.4%)	24 (2.0%)	0 (0.0%)
Pediatric Hematology and Oncology	3 (0.2%)	3 (0.2%)	0 (0.0%)
Pediatric Infectious Diseases	1 (0.1%)	1 (0.1%)	0 (0.0%)
Pediatric Inpatient Team (General)	300 (17.8%)	300 (25.0%)	0 (0.0%)
Pediatric Nephrology	2 (0.1%)	2 (0.2%)	0 (0.0%)
Pediatric Neurology	3 (0.2%)	3 (0.2%)	0 (0.0%)
Pediatric Neurosurgery	4 (0.2%)	4 (0.3%)	0 (0.0%)
Pediatric Optometry and Ophthalmology	3 (0.2%)	3 (0.2%)	0 (0.0%)
Pediatric Organ Transplant & Hepatobiliary Surgery	3 (0.2%)	3 (0.2%)	0 (0.0%)
Pediatric Orthopedic	1 (0.1%)	1 (0.1%)	0 (0.0%)
Pediatric Plastic Surgery	9 (0.5%)	9 (0.7%)	0 (0.0%)
Pediatric Pulmonology	2 (0.1%)	2 (0.2%)	0 (0.0%)
Pediatric Rheumatology	4 (0.2%)	4 (0.3%)	0 (0.0%)
Pediatric Surgery	10 (0.6%)	10 (0.8%)	0 (0.0%)
Pediatric Urology	1 (0.1%)	1 (0.1%)	0 (0.0%)
PICU	2 (0.1%)	2 (0.2%)	0 (0.0%)
Pulmonology	1 (0.1%)	0 (0.0%)	1 (0.2%)
Transplant Nephrology	1 (0.1%)	0 (0.0%)	1 (0.2%)

[1]n (%); Median (Q1 - Q3)

Table S2. Number of visits per year (n=27902).

Characteristic	2019	2020	2021	2022	2023	2024
**Pediatric (<18 years)**						
Number of visits						
1	4,374 (80.4%)	1,793 (87.6%)	1,975 (86.5%)	3,510 (83.1%)	3,944 (80.4%)	3,182 (80.2%)
2	773 (14.2%)	198 (9.7%)	252 (11.0%)	533 (12.6%)	668 (13.6%)	539 (13.6%)
3	202 (3.7%)	28 (1.4%)	42 (1.8%)	136 (3.2%)	197 (4.0%)	173 (4.4%)
4	62 (1.1%)	16 (0.8%)	8 (0.4%)	31 (0.7%)	57 (1.2%)	55 (1.4%)
5	15 (0.3%)	8 (0.4%)	3 (0.1%)	10 (0.2%)	25 (0.5%)	9 (0.2%)
6	5 (0.1%)	2 (0.1%)	1 (0.0%)	4 (0.1%)	4 (0.1%)	5 (0.1%)
7	5 (0.1%)	0 (0.0%)	1 (0.0%)	0 (0.0%)	5 (0.1%)	0 (0.0%)
8	0 (0.0%)	0 (0.0%)	1 (0.0%)	1 (0.0%)	2 (0.0%)	1 (0.0%)
9	1 (0.0%)	1 (0.0%)	0 (0.0%)	1 (0.0%)	1 (0.0%)	2 (0.1%)
10	0 (0.0%)	0 (0.0%)	0 (0.0%)	0 (0.0%)	1 (0.0%)	2 (0.1%)
12	0 (0.0%)	0 (0.0%)	0 (0.0%)	0 (0.0%)	1 (0.0%)	0 (0.0%)
Number of visits						
Once	4,374 (80.4%)	1,793 (87.6%)	1,975 (86.5%)	3,510 (83.1%)	3,944 (80.4%)	3,182 (80.2%)
Twice or more	1,063 (19.6%)	253 (12.4%)	308 (13.5%)	716 (16.9%)	961 (19.6%)	786 (19.8%)
**Adult (≥18 years)**						
Number of visits						
1	1,055 (92.1%)	622 (90.9%)	665 (90.4%)	623 (89.5%)	832 (89.9%)	773 (90.5%)
2	75 (6.6%)	49 (7.2%)	58 (7.9%)	60 (8.6%)	80 (8.6%)	67 (7.8%)
3	11 (1.0%)	9 (1.3%)	7 (1.0%)	8 (1.1%)	9 (1.0%)	8 (0.9%)
4	1 (0.1%)	2 (0.3%)	4 (0.5%)	3 (0.4%)	2 (0.2%)	4 (0.5%)
5	3 (0.3%)	0 (0.0%)	1 (0.1%)	2 (0.3%)	1 (0.1%)	0 (0.0%)
6	0 (0.0%)	2 (0.3%)	0 (0.0%)	0 (0.0%)	0 (0.0%)	1 (0.1%)
7	0 (0.0%)	0 (0.0%)	1 (0.1%)	0 (0.0%)	0 (0.0%)	0 (0.0%)
9	0 (0.0%)	0 (0.0%)	0 (0.0%)	0 (0.0%)	1 (0.1%)	0 (0.0%)
10	0 (0.0%)	0 (0.0%)	0 (0.0%)	0 (0.0%)	0 (0.0%)	1 (0.1%)
Number of visits						
Once	1,055 (92.1%)	622 (90.9%)	665 (90.4%)	623 (89.5%)	832 (89.9%)	773 (90.5%)
Twice or more	90 (7.9%)	62 (9.1%)	71 (9.6%)	73 (10.5%)	93 (10.1%)	81 (9.5%)

[1]n (%)

Table S3. Frequencies and percentages of visits by ICD-10 codes.

Characteristic	**Overall** N = 34,290[1]	**Pediatric (<18 years)** N=28643[1]	**Adult (≥18 years)** N=5647[1]
Internal Diagnosis			
Acute pharyngitis - unspecified	9,980 (29.1%)	9,202 (32.1%)	778 (13.8%)
Acute tonsillitis - unspecified	8,971 (26.2%)	7,699 (26.9%)	1,272 (22.5%)
Otitis media - unspecified	7,654 (22.3%)	7,136 (24.9%)	518 (9.2%)
Epistaxis	1,830 (5.3%)	825 (2.9%)	1,005 (17.8%)
Acute otitis media	621 (1.8%)	567 (2.0%)	54 (1.0%)
Acute tonsillitis	276 (0.8%)	266 (0.9%)	10 (0.2%)
Acute sinusitis - unspecified	223 (0.7%)	84 (0.3%)	139 (2.5%)
Acute pharyngitis	161 (0.5%)	157 (0.5%)	4 (0.1%)
Acute pharyngitis due to other specified organisms	69 (0.2%)	50 (0.2%)	19 (0.3%)
Acute tonsillitis due to other specified organisms	65 (0.2%)	38 (0.1%)	27 (0.5%)
Abscess of fasciaother site	1 (0.0%)	0 (0.0%)	1 (0.0%)
Acute laryngitis	18 (0.1%)	9 (0.0%)	9 (0.2%)
Acute laryngotracheitis	14 (0.0%)	14 (0.0%)	0 (0.0%)
Acute noninfective otitis externa,other	1 (0.0%)	1 (0.0%)	0 (0.0%)
Acute otitis externa,noninfective	7 (0.0%)	7 (0.0%)	0 (0.0%)
Acute rhinitis	2 (0.0%)	2 (0.0%)	0 (0.0%)
Acute serous otitis media	13 (0.0%)	9 (0.0%)	4 (0.1%)
Acute suppurative otitis media	58 (0.2%)	51 (0.2%)	7 (0.1%)
Acute suppurative otitis media with eardrum rupture	3 (0.0%)	2 (0.0%)	1 (0.0%)
Acute suppurative otitis media without eardrum rupture	3 (0.0%)	2 (0.0%)	1 (0.0%)
Acute upper respiratory infection - unspecified	5 (0.0%)	4 (0.0%)	1 (0.0%)
Allergic reaction	1 (0.0%)	0 (0.0%)	1 (0.0%)
Anaphylactic shock - unspecified	1 (0.0%)	0 (0.0%)	1 (0.0%)
Anaphylaxis	2 (0.0%)	0 (0.0%)	2 (0.0%)
Candidal otitis externa	1 (0.0%)	0 (0.0%)	1 (0.0%)
Central perforation of tympanic membrane	1 (0.0%)	0 (0.0%)	1 (0.0%)
Chronic atticoantral suppurative otitis media	1 (0.0%)	0 (0.0%)	1 (0.0%)
Chronic otitis externa	1 (0.0%)	1 (0.0%)	0 (0.0%)
Chronic otitis media	16 (0.0%)	8 (0.0%)	8 (0.1%)
Chronic otitis media with cholesteatoma	4 (0.0%)	4 (0.0%)	0 (0.0%)
Chronic serous otitis media	3 (0.0%)	0 (0.0%)	3 (0.1%)
Chronic sinusitis - unspecified	98 (0.3%)	11 (0.0%)	87 (1.5%)
Chronic tonsillitis	2 (0.0%)	0 (0.0%)	2 (0.0%)
Chronic tubotympanic suppurative otitis media	12 (0.0%)	3 (0.0%)	9 (0.2%)
Conductive hearing loss,bilateral	2 (0.0%)	1 (0.0%)	1 (0.0%)
Cough	1 (0.0%)	1 (0.0%)	0 (0.0%)
Dental infection	1 (0.0%)	0 (0.0%)	1 (0.0%)
Deviated nasal septum	168 (0.5%)	19 (0.1%)	149 (2.6%)
Diffuse otitis externa	1 (0.0%)	1 (0.0%)	0 (0.0%)
Disorder of tympanic membrane - unspecified	1 (0.0%)	1 (0.0%)	0 (0.0%)
Dysphagia	304 (0.9%)	51 (0.2%)	253 (4.5%)
Foreign body in ear	383 (1.1%)	329 (1.1%)	54 (1.0%)
Foreign body in nose	418 (1.2%)	416 (1.5%)	2 (0.0%)
Foreign body in nostril	221 (0.6%)	220 (0.8%)	1 (0.0%)
Foreign body in pharynx	36 (0.1%)	21 (0.1%)	15 (0.3%)
Foreign body in throat	83 (0.2%)	37 (0.1%)	46 (0.8%)

Functional hearing loss	2 (0.0%)	2 (0.0%)	0 (0.0%)
Gastroenteritis	1 (0.0%)	1 (0.0%)	0 (0.0%)
Haemorrhoids - unspecified	1 (0.0%)	0 (0.0%)	1 (0.0%)
Hearing loss - unspecified	39 (0.1%)	14 (0.0%)	25 (0.4%)
Impacted cerumen	34 (0.1%)	23 (0.1%)	11 (0.2%)
Injury of arm	1 (0.0%)	0 (0.0%)	1 (0.0%)
Insect bite of lower leg	1 (0.0%)	1 (0.0%)	0 (0.0%)
Knee pain	1 (0.0%)	0 (0.0%)	1 (0.0%)
Malignant otitis externa	8 (0.0%)	0 (0.0%)	8 (0.1%)
Medical care - unspecified	5 (0.0%)	0 (0.0%)	5 (0.1%)
Mixed conductive and sensorineural hearing loss,bilateral	1 (0.0%)	0 (0.0%)	1 (0.0%)
Mixed hearing loss	1 (0.0%)	1 (0.0%)	0 (0.0%)
Noise-induced hearing loss	1 (0.0%)	0 (0.0%)	1 (0.0%)
Nonsuppurative otitis media	3 (0.0%)	3 (0.0%)	0 (0.0%)
Nonsuppurative otitis media - unspecified	26 (0.1%)	18 (0.1%)	8 (0.1%)
Other acute nonsuppurative otitis media	123 (0.4%)	121 (0.4%)	2 (0.0%)
Other acute sinusitis	30 (0.1%)	14 (0.0%)	16 (0.3%)
Other chronic nonsuppurative otitis media	2 (0.0%)	1 (0.0%)	1 (0.0%)
Other chronic sinusitis	2 (0.0%)	0 (0.0%)	2 (0.0%)
Other chronic suppurative otitis media	24 (0.1%)	14 (0.0%)	10 (0.2%)
Other conjunctivitis	1 (0.0%)	0 (0.0%)	1 (0.0%)
Other infective otitis externa	3 (0.0%)	3 (0.0%)	0 (0.0%)
Other otitis externa	95 (0.3%)	68 (0.2%)	27 (0.5%)
Other perforations of tympanic membrane	2 (0.0%)	1 (0.0%)	1 (0.0%)
Other specified disorders of tympanic membrane	5 (0.0%)	4 (0.0%)	1 (0.0%)
Other specified hearing loss	2 (0.0%)	2 (0.0%)	0 (0.0%)
Otitis externa	3 (0.0%)	1 (0.0%)	2 (0.0%)
Otitis externa - unspecified	914 (2.7%)	399 (1.4%)	515 (9.1%)
Otitis externa in bacterial disease classified elsewhere	1 (0.0%)	0 (0.0%)	1 (0.0%)
Otitis externa in bacterial diseases classified elsewhere	2 (0.0%)	0 (0.0%)	2 (0.0%)
Otitis externa in mycoses	2 (0.0%)	0 (0.0%)	2 (0.0%)
Otitis externa in mycosis	11 (0.0%)	2 (0.0%)	9 (0.2%)
Otitis externa in other diseases classified elsewhere	3 (0.0%)	0 (0.0%)	3 (0.1%)
Otitis media	104 (0.3%)	103 (0.4%)	1 (0.0%)
Otitis media in bacterial diseases classified elsewhere	7 (0.0%)	7 (0.0%)	0 (0.0%)
Otitis media in influenza	7 (0.0%)	7 (0.0%)	0 (0.0%)
Otitis media in other disease classified elsewhere	1 (0.0%)	0 (0.0%)	1 (0.0%)
Otitis media in other diseases classified elsewhere	3 (0.0%)	3 (0.0%)	0 (0.0%)
Otitis media in scarlet fever	3 (0.0%)	3 (0.0%)	0 (0.0%)
Otitis media in viral diseases classified elsewhere	6 (0.0%)	5 (0.0%)	1 (0.0%)
Otitis media with effusion	154 (0.4%)	133 (0.5%)	21 (0.4%)
Pain - unspecified	9 (0.0%)	0 (0.0%)	9 (0.2%)
Pain in a joint,ankle and foot	1 (0.0%)	0 (0.0%)	1 (0.0%)
Perforation of tympanic membrane - unspecified	16 (0.0%)	11 (0.0%)	5 (0.1%)
Peritonsillar abscess	31 (0.1%)	14 (0.0%)	17 (0.3%)
Postoperative bleeding after tonsillectomy	2 (0.0%)	2 (0.0%)	0 (0.0%)
Sensorineural hearing loss - unspecified	1 (0.0%)	1 (0.0%)	0 (0.0%)
Sensorineural hearing loss,bilateral	5 (0.0%)	5 (0.0%)	0 (0.0%)
Sensory neural hearing loss,unilateral with normal hearing on the other side	1 (0.0%)	0 (0.0%)	1 (0.0%)
Sideropenic dysphagia	1 (0.0%)	0 (0.0%)	1 (0.0%)
Sinusitis	559 (1.6%)	220 (0.8%)	339 (6.0%)

Sprain and strain of joints and ligaments of other and unspecified parts of neck	1 (0.0%)	0 (0.0%)	1 (0.0%)
Submandibular abscess	1 (0.0%)	0 (0.0%)	1 (0.0%)
Sudden idiopathic hearing loss	1 (0.0%)	0 (0.0%)	1 (0.0%)
Superficial injury of foreheadunspecified	1 (0.0%)	1 (0.0%)	0 (0.0%)
Suppurative otitis media - unspecified	91 (0.3%)	82 (0.3%)	9 (0.2%)
Throat pain	165 (0.5%)	85 (0.3%)	80 (1.4%)
Tympanic membrane perforation,traumatic	32 (0.1%)	19 (0.1%)	13 (0.2%)

[1]n (%)

Table S4: Frequencies and percentages of visits by ICD-10 codes (n=34290)

Characteristic	Description, N=34290
ICD	
A	4 (0.0%)
B	1 (0.0%)
D	1 (0.0%)
F	2 (0.0%)
H	10,098 (29.4%)
J	20,681 (60.3%)
K	3 (0.0%)
M	3 (0.0%)
R	2,309 (6.7%)
S	36 (0.1%)
T	1,147 (3.3%)
Z	5 (0.0%)

Table S5. Baseline characteristics of patients who visited once versus those who visited twice or more (n=22014).

**Characteristic**	**Once** N=15293[1]	**Twice or more** N=6721[1]	**p-value**[2]
Month of visit			<0.001
January	1,474 (66.2%)	753 (33.8%)	
February	1,448 (65.9%)	749 (34.1%)	
March	1,336 (63.7%)	760 (36.3%)	
April	1,119 (67.6%)	536 (32.4%)	
May	1,160 (68.3%)	538 (31.7%)	
June	1,132 (71.6%)	448 (28.4%)	
July	916 (67.5%)	441 (32.5%)	
August	1,010 (71.4%)	404 (28.6%)	
September	1,205 (72.3%)	462 (27.7%)	
October	1,607 (72.2%)	620 (27.8%)	
November	1,505 (73.5%)	543 (26.5%)	
December	1,381 (74.7%)	467 (25.3%)	
Day of visit			0.706
Saturday	2,345 (70.1%)	998 (29.9%)	
Sunday	2,218 (69.3%)	983 (30.7%)	
Monday	2,071 (69.5%)	911 (30.5%)	
Tuesday	2,173 (68.7%)	991 (31.3%)	
Wednesday	2,152 (69.1%)	963 (30.9%)	
Thursday	2,090 (70.6%)	872 (29.4%)	
Friday	2,244 (69.1%)	1,003 (30.9%)	
ER Check In Time			0.068
Morning (06:00 to 11:59)	3,252 (70.4%)	1,365 (29.6%)	
Afternoon (12:00 to 16:59)	3,438 (70.4%)	1,447 (29.6%)	
Evening (17:00 to 20:59)	3,290 (68.5%)	1,512 (31.5%)	
Night (21:00 to 5:59)	5,313 (68.9%)	2,397 (31.1%)	

[1]n (%)

[2]Pearson's Chi-squared test; Fisher's exact test
